# Dissemination of KPC-2-Encoding IncX6 Plasmids Among Multiple *Enterobacteriaceae* Species in a Single Chinese Hospital

**DOI:** 10.3389/fmicb.2018.00478

**Published:** 2018-03-19

**Authors:** Bing Li, Jiao Feng, Zhe Zhan, Zhe Yin, Qiyu Jiang, Ping Wei, Xingming Chen, Bo Gao, Jun Hou, Panyong Mao, Weili Wu, Weijun Chen, Yigang Tong, Jinglin Wang, Boan Li, Dongsheng Zhou

**Affiliations:** ^1^Department of Clinical Laboratory, The 306th Hospital of the People’s Liberation Army, Beijing, China; ^2^State Key Laboratory of Pathogen and Biosecurity, Beijing Institute of Microbiology and Epidemiology, Beijing, China; ^3^The 302nd Hospital of the People’s Liberation Army, Beijing, China; ^4^Beijing Institute of Genomics, Chinese Academy of Sciences, Beijing, China

**Keywords:** plasmid, IncX6, genomics, epidemiology, *bla*_KPC_

## Abstract

Forty-five KPC-producing *Enterobacteriaceae* strains were isolated from multiple departments in a Chinese public hospital from 2014 to 2015. Genome sequencing of four representative strains, namely *Proteus mirabilis* GN2, *Serratia marcescens* GN26, *Morganella morganii* GN28, and *Klebsiella aerogenes* E20, indicated the presence of *bla*_KPC-2_-carrying IncX6 plasmids pGN2-KPC, pGN26-KPC, pGN28-KPC, and pE20-KPC in the four strains, respectively. These plasmids were genetically closely related to one another and to the only previously sequenced IncX6 plasmid, pKPC3_SZ. Each of the plasmids carried a single accessory module containing the *bla*_KPC-2/3_-carrying ΔTn*6296* derivatives. The ΔTn*6292* element from pGN26-KPC also contained *qnrS*, which was absent from all other plasmids. Overall, pKPC3_SZ-like *bla*_KPC_-carrying IncX6 plasmids were detected by PCR in 44.4% of the KPC-producing isolates, which included *K. aerogenes, P. mirabilis, S. marcescens, M. morganii, Escherichia coli*, and *Klebsiella pneumoniae*, and were obtained from six different departments of the hospital. Data presented herein provided insights into the genomic diversity and evolution of IncX6 plasmids, as well as the dissemination and epidemiology of *bla*_KPC_-carrying IncX6 plasmids among *Enterobacteriaceae* in a hospital setting.

## Introduction

*Klebsiella pneumoniae* carbapenamase (KPC), a class A β-lactamase, can hydrolyze almost all β-lactams, including carbapenems (Bush and Fisher, 2011). At least 31 variants (KPC-2 to KPC-32; KPC-1 is essentially identical to KPC-2) of the KPC enzyme have been identified to date^1^. Two Tn*3*-family unit transposons, Tn*4401* and Tn*6296*, which are genetically divergent from each other, represent the two major prototype genetic platforms carrying *bla*_KPC_ genes (Wang et al., 2015). Tn*4401* and its derivatives are frequently identified in KPC-encoding plasmids of different incompatibility groups in bacterial isolates from European and American countries, but are rarely found in isolates from China (Feng et al., 2017). The *bla*_KPC_ genetic environment in isolates from China is predominantly associated with Tn*6296* and its derivatives (Wang et al., 2015).

Plasmids belonging to incompatibility group X (IncX) are 30–80 kb in size and were initially discovered in the pre-antibiotic era (Datta and Hughes, 1983). IncX plasmids have a narrow host range and are mainly circulated among *Enterobacteriaceae* species (Norman et al., 2008). The backbones of all IncX plasmids have a *pir*–*parA*–*hns*–*hha*–*topB*–*pilX* (*tivB*)–*actX*–*taxC* (*rlx*)–*taxA* (*dtr*) organization, but are quite divergent with respect to nucleotide and amino acid sequences similarity (Johnson et al., 2012). Comparative genomic analysis has shown that IncX plasmids can be phylogenetically grouped into seven major IncX subgroups, IncX1 to IncX6 (Du et al., 2016), along with another IncX6 subgroup (Bustamante and Iredell, 2017) that is re-designated herein IncX7.

*bla*_KPC-2_-harboring plasmids have been identified among the IncX3, IncX5, and IncX6 subgroups, including over a dozen plasmids belonging to subgroup IncX3 [e.g., pKpS90 (GenBank accession number JX461340) (Kassis-Chikhani et al., 2013) and pMNCRE44_5 (GenBank accession number CP010881) (Hargreaves et al., 2015)], two IncX5 plasmids [pKPC_CAV1492 (GenBank accession number CP011639) and pBK31567 (GenBank accession number JX193302) (Chen et al., 2013)], and a single IncX6 plasmid [pKPC3_SZ (GenBank accession number KU302800) (Du et al., 2016)]. Interestingly, *bla*_KPC-2_-harboring plasmids have not been found among the other IncX subgroups.

This study provides evidence for the dissemination of genetically highly similar KPC-2-encoding IncX6 plasmids among at least six *Enterobacteriaceae* species collected in a Chinese public hospital from 2014 to 2015. The complete nucleotide sequences of plasmids pGN2-KPC, pGN26-KPC, pGN28-KPC, and pE20-KPC, extracted from strains belonging to four representative species, were determined to be genetically closely related to the IncX6 reference plasmid pKPC3_SZ. In addition, all five plasmids carried a single accessory region that harbored the *bla*_KPC-2/3_ gene.

## Materials and Methods

### Bacterial Identification

Bacterial species identification was performed on the basis of 16S rRNA gene sequencing (Frank et al., 2008). The major plasmid-borne carbapenemase and extended-spectrum β-lactamase genes were screened for by PCR (Chen et al., 2014). All PCR amplicons were sequenced using an ABI 3730 Sequencer (Life Technologies, Carlsbad, CA, United States) using the primers used for PCR.

### Plasmid Transfer

Plasmid conjugal transfer experiments were carried out using rifampin-resistant *Escherichia coli* strain EC600 (LacZ-, NalR, RifR) as the recipient and each of *Proteus mirabilis* GN2, *Serratia marcescens* GN26, *Morganella morganii* GN28, and *Klebsiella aerogenes* E20 as the donor (Feng et al., 2016). Aliquots (3 ml) of overnight cultures of each of the donor and recipient strains were mixed together, harvested, and resuspended in 80 μl of brain heart infusion broth (BD Biosciences, Franklin Lakes, NJ, United States). The mixture was spotted onto a 1-cm^2^ hydrophilic nylon membrane filter with a 0.45-μm pore size (Millipore, Billerica, MA, United States) placed onto the surface of a brain heart infusion agar (BD Biosciences, Franklin Lakes, NJ, United States) plate. Plates were incubated for mating at 37°C for 12–18 h. Bacteria were washed from the filter membrane and spotted on Mueller-Hinton agar (BD Biosciences, Franklin Lakes, NJ, United States) plates containing 1 mg/ml rifampin and 2 μg/ml imipenem to select the transconjugants containing the *bla*_KPC_ marker.

### Phenotypic Assays

Activity of Ambler class A/B/D carbapenemases in bacterial cell extracts was determined by a modified CarbaNP test (Feng et al., 2016). Bacterial antimicrobial susceptibility was examined using the broth dilution method, and interpreted as per the Clinical and Laboratory Standards Institute guidelines (CLSI, 2015).

### Genomic DNA Sequencing and Sequence Assembly

Genomic DNA was isolated from *Enterobacteriaceae* isolates GN2, GN26, GN28, and E20 using a Blood and Cell Culture DNA Maxi Kit (Qiagen, Hilden, Germany). Genome sequencing was performed for isolate GN2 using a sheared DNA library with an average size of 15 kb (ranging from 10 to 20 kb) on a PacBio RSII sequencer (Pacific Biosciences, Menlo Park, CA, United States), as well as with a paired-end library with an average insert size of 400 bp (ranging from 150 to 600 kb) on a HiSeq sequencer (Illumina, San Diego, CA, United States). The paired-end short Illumina reads were used to correct the long PacBio reads using *proovread* (Hackl et al., 2014), then the corrected PacBio reads were assembled *de novo* using SMARTdenovo^2^.

Genomic DNA from isolates GN26, GN28, and E20 was sequenced from a mate-pair libraries with an average insert size of 5 kb (ranging from 2 to 10 kb) using a MiSeq sequencer (Illumina, San Diego, CA, United States). DNA contigs that were not matched with the reference chromosome sequences of *S. marcescens* (GenBank accession number HG738868), *M. morganii* (GenBank accession number CP023505) or *K. aerogenes* (GenBank accession number FO203355) were assembled based on their contig coverage values using Newbler 2.6 (Nederbragt, 2014). Gaps between contigs were filled using a combination of PCR and Sanger sequencing using an ABI 3730 Sequencer.

### Sequence Annotation and Genome Comparison

Open reading frames (ORFs) and pseudogenes were predicted using RAST 2.0 (Brettin et al., 2015) combined with BLASTP/BLASTN searches (Boratyn et al., 2013) against the UniProtKB/Swiss-Prot database (Boutet et al., 2016) and the RefSeq database (O’Leary et al., 2016). Resistance genes, mobile elements, and other features were annotated using online databases including CARD (Jia et al., 2017), ResFinder (Zankari et al., 2012), ISfinder (Siguier et al., 2006), and the Tn Number Registry (Roberts et al., 2008). Multiple and pairwise sequence comparisons were performed using MUSCLE 3.8.31 (Edgar, 2004) and BLASTN, respectively. Gene organization diagrams were drawn in Inkscape 0.48.1^3^.

### Nucleotide Sequence Accession Numbers

The complete sequences of plasmids pE20-KPC, pGN2-KPC, pGN26-KPC, and pGN28-KPC and the draft sequences of the E20, GN2, GN26, and GN28 chromosomes were submitted to GenBank under accession numbers MF156709 to MF156712, CP026722, CP026581, CP026650, and CP026651, respectively.

## Results

### *bla*_KPC_-Carrying Isolates

From 2014 to 2015, a total of 143 carbapenem-resistant *Alcaligenes xylosoxidans* (*n* = 6), *Acinetobacter baumannii* (*n* = 24), *Pseudomonas aeruginosa* (*n* = 47), *Pseudomonas putida* (*n* = 5), *K. aerogenes* (*n* = 6), *E. coli* (*n* = 2), *Enterobacter cloacae* (*n* = 2), *K. pneumoniae* (*n* = 35), *P. mirabilis* (*n* = 8), *S. marcescens* (*n* = 5), and *M. morganii* (*n* = 3) isolates were obtained from the 143 different patients with various infections at a Chinese public hospital. Of these carbapenem-resistant isolates, 45 (31.5%) demonstrated class A carbapenemase activity and contained *bla*_KPC_ genes, while three isolates (2.1%) had class B carbapenemase activity and carried *bla*_NDM_ genes. Carbapenemase activity and major plasmid-borne carbapenemase genes were not detected in the remaining strains (66.4%). All of the carbapenemase-positive isolates were identified as *Enterobacteriaceae*.

These 45 *bla*_KPC_-carrying isolates consisted of *K. pneumonia* (*n* = 31), *K. aerogenes* (*n* = 6), *S. marcescens* (*n* = 5), and one isolate each of *M. morganii, E. coli*, and *P. mirabilis*. In total, 36 of the isolates were recovered from sputum specimens, while the remaining isolates were obtained from urine specimens. The 45 isolates came from 10 different hospital departments: 25 from the Intensive Care Unit, 6 from the Department of Gerontology, 5 from the Department of Respiratory Medicine, 2 from the Department of Neurology, 2 from the Department of Urology, and 1 each from the Department of General Surgery, the Department of Neurosurgery, the Emergency Department, the Department of Endocrinology, and the Department of Traditional Chinese Medicine (**Supplementary Table S1**). Thirty-six (80.0%) of the 45 isolates carried one or more β-lactamase genes [*bla*_TEM_, *bla*_SHV_, *bla*_CTX-M-1G (Group)_, *bla*_CTX-M-9G_, *bla*_OXA-1_ and *bla*_OXA-2_] in addition to *bla*_KPC_.

### pKPC3_SZ-Like IncX6 Plasmids From *bla*_KPC-2_-Carrying Isolates

Four *bla*_KPC_-positive isolates, *P. mirabilis* GN2, *S. marcescens* GN26, *M. morganii* GN28, and *K. aerogenes* E20, were arbitrarily selected for genome sequencing. GN2 was isolated from the urine specimens of an elderly female with urinary tract infection in 2014, while GN26 and GN28 (in 2015) and E20 (in 2014) were isolated from sputum specimens from three different elderly males suffering from pulmonary infections. These four patients were admitted to the hospital because of primary diseases consisting of myocardial infarction, cerebral infarction sequelae, cerebral contusion and pneumonia, respectively, and developed the above hospital-acquired infections during hospitalization.

**FIGURE 1:**
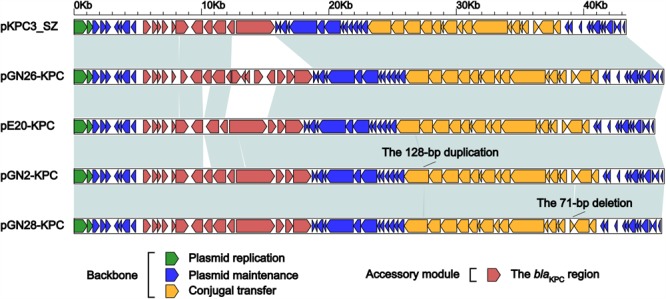
Linear comparison of IncX6 plasmids. Genes are denoted by arrows. Genes, mobile elements and other features are colored based on function classification. Shading denotes regions of homology (>95% nucleotide identity).

GN2, GN26, GN28, and E20 each contained an IncX6 plasmid, designated pGN2-KPC, pGN26-KPC, pGN28-KPC, and pE20-KPC, respectively. These plasmids were 45.6–46.3 kb in length, with 62–65 predicted ORFs (**Table 1**). The modular structure of each plasmid was divided into the backbone regions along with a single accessory module, which was defined as an acquired DNA region associated with mobile elements, and was inserted into the backbone (**Figure 1** and **Supplementary Figure S1**). A total of three resistance genes were identified: *bla*_KPC-2_ was located in all four plasmids, while *Δbla*_TEM-1_ was identified in pGN2-KPC, pGN26-KPC, and pGN28-KPC, and *qnrS1* was found in pGN26-KPC. All these resistance genes were located in the accessory modules.

**Table 1:** Major features of plasmids analyzed.

Category	IncX6 plasmids				
	pGN2-KPC	pGN26-KPC	pGN28-KPC	pE20-KPC	pKPC3_SZ
Total length (bp)	46,320	46,292	46,123	45,579	43,333
Total number of ORFs	65	65	63	62	61
Mean G+C content, %	47.9	47.7	47.9	47.9	47.7
Length of the backbone (bp)	32,849	32,720	32,652	32,722	32,721
Accessory module(s) [resistance gene(s) harbored]	The *bla*_KPC-2_ region (*bla*_KPC-2_ and *Δbla*_TEM-1_)	The *bla*_KPC-2_ region (*bla*_KPC-2_, *Δbla*_TEM-1_ and *qnrS1*)	The *bla*_KPC-2_ region (*bla*_KPC-2_ and *Δbla*_TEM-1_)	The *bla*_KPC-2_ region (*bla*_KPC-2_)	The *bla*_KPC-3_ region (*bla*_KPC-3_ and *Δbla*_TEM-1_)

*pGN2-KPC, pGN26-KPC, pGN28-KPC, and pE20-KPC were fully sequenced in this work, while pKPC3_SZ was derived from GenBank. Genetic comparison of these five sequenced plasmids was interpreted in the main text.*

All four *bla*_KPC-2_-carrying plasmids could be transferred to *E. coli* EC600 via conjugation, generating the corresponding *bla*_KPC_-positive *E. coli* transconjugants GN2-KPC-EC600, GN26-KPC-EC600, GN28-KPC-EC600, and E20-KPC-EC600. Class A carbapenemase activity was detected for all transconjugants, and resulted from the production of the KPC-2 enzyme. Both the wild-type and transconjugant strains were resistant to ampicillin, cefepime, meropenem, and aztreonam. Moreover, GN26 was resistant to ciprofloxacin, but its transconjugant was intermediately resistant to this drug due to the presence of *qnrS1* known to mediate the low-level resistance to fluoroquinolones (**Table 2**). In conclusion, each of GN2, GN26, GN28, and E20 harbored a conjugative *bla*_KPC_-carrying IncX6 plasmid, which accounted for the carbapenem resistance phenotype.

**Table 2:** Antimicrobial drug susceptibility profiles.

Antibiotics	MIC (mg/L)/antimicrobial susceptibility								
	GN2	GN2-KPC -EC600	GN26	GN26-KPC -EC600	GN28	GN28-KPC -EC600	E20	E20-KPC -EC600	EC600
Ampicillin	>1024/R	>1024/R	>1024/R	>1024/R	>1024/R	>1024/R	>1024/R	>1024/R	<4/S
Cefepime	>256/R	64/R	>256/R	16/R	256/R	128/R	>256/R	32/R	<2/S
Meropenem	16/R	4/R	32/R	8/R	16/R	4/R	32/R	8/R	<1/S
Aztreonam	>512/R	512/R	>512/R	>512/R	>512/R	>512/R	>512/R	>512/R	<4/S
Amikacin	<8/S	<8/S	<8/S	<8/S	<8/S	<8/S	>1024/R	<8/S	<8/S
Tetracycline	64/R	<1/S	64/R	<1/S	64/R	<1/S	64/R	<1/S	<1/S
Ciprofloxacin	16/R	<1/S	4/R	2/I	64/R	<1/S	128/R	<1/S	<1/S
Nitrofurantoin	128/R	16/S	>128/R	16/S	64/I	16/S	128/R	16/S	8/S
Trimethoprim	>32/R	<0.25/S	<0.25/S	<0.25/S	<0.25/S	<0.25/S	>32/R	<0.25/S	<0.25/S
Sulfamethoxazole	>608/R	<4.75/S	<4.75/S	<4.75/S	<4.75/S	<4.75/S	>608/R	<4.75/S	<4.75/S
Tigecycline	<1/S	<1/S	<1/S	<1/S	<1/S	<1/S	<1/S	<1/S	<1/S
Colistin	128/R	<1/S	>128/R	<1/S	<1/S	<1/S	<1/S	<1/S	<1/S

*S, sensitive; R, resistant; I, intermediately resistant.*

Based on the complete sequences of the five IncX6 plasmids (pGN2-KPC, pGN26-KPC, pGN28-KPC, pE20-KPC, and pKPC3_SZ), a total of nine genes were arbitrarily selected to screen for the prevalence of pKPC3_SZ-like IncX6 plasmids among the 45 *bla*_KPC_-positive isolates. Of these nine selected genes, eight [replication initiation: *repA* (replication initiation protein); maintenance: *parA* (partitioning ATPase), *topB* (type III topoisomerase), *dnaJ* (molecular chaperone), and *ftsH* (cell division protein); conjugal transfer: *tivB3-4* (P-type type IV secretion, inner-membrane component of translocation channel and ATPase), *tivB6* (P-type type IV secretion, inner-membrane component of translocation channel), and *tivB10* (P-type type IV secretion, outer-membrane component of translocation channel)] were from backbone regions, while the remaining one was quinolone-resistance gene *qnrS1*. PCR analysis and amplicon sequencing showed that all eight backbone genes were present in 24 isolates, including 11 *K. pneumoniae* isolates, all 6 *K. aerogenes* isolates, 4 *S. marcescens* isolates, and 1 isolate each of *M. morganii, E. coli*, and *P. mirabilis*, indicating that these isolates harbored IncX6 plasmids. All five replication and maintenance genes, but none of the three conjugal transfer genes, were detected in another *S. marcescens* isolate, probably indicating that this isolate contained an IncX6 plasmid that had lost the conjugal transfer genes. None of the eight selected genes were detected in the remaining 20 isolates, signifying that these isolates did not carry IncX6 plasmids. *qnrS1* was detected in 26 *K. pneumoniae* isolates, 6 *K. aerogenes* isolates, and 4 *S. marcescens* isolates, denoting coexistence of *bla*_KPC_ and *qnrS1* in these isolates.

### Genomic Comparison of IncX6 Plasmids

pGN2-KPC, pGN26-KPC, pGN28-KPC, and pE20-KPC showed the highest sequence identity to the IncX6 reference plasmid pKPC3_SZ (Du et al., 2016), with >92% query coverage and >99% nucleotide identity. The major backbone genes or gene loci included *repA* and its iterons (replication initiation), *parA* and *topB*–*hha*–*hns* (maintenance), and *rlx, dtr, tivB, cpl*, and *eex* (conjugal transfer). *repA* coded for the IncX6-specific replication initiation protein and was not identified in any other available sequences. A 253-bp region containing seven imperfect GGTTTTTAAATCCCGata direct repeats was located 73-bp upstream of *repA*, and may function as iterons that bind the RepA protein. ParA was the partitioning ATPase responsible for plasmid segregation and stability (Schumacher, 2008), however, centromere-binding protein ParB and its binding sites *parC* could be not located. The gene expression modulation (*gem*) region, composed of *topB, hha* (transcriptional regulator), and *hns* (histone-like DNA-binding protein), was involved in plasmid maintenance (Norman et al., 2008). The conjugal transfer region was composed of a complete set of P-type conjugative DNA transfer genes, including *rlx* and *dtr* (DNA transfer; encoding relaxase Rlx and an auxiliary protein, Dtr), *tivB1–tivB11* (encoding P-type type IV secretion system elaborating the pilus for mating pair formation), *cpl* (encoding a coupling protein that links DNA transfer and mating pair formation), and *eex* (entry exclusion preventing nucleoprotein transport between donors) (De La Cruz et al., 2010; Chen et al., 2013; Thomas et al., 2017).

The backbones of these five plasmids displayed only two major modular differences (**Figure 1**): (i) a 128-bp duplication in *cpl* of pGN2-KPC resulted in frameshift mutation, turning *cpl* into a pseudogene but retaining the conjugal transfer ability of pGN2-KPC, and (ii) a 71-bp deletion within *orf393* (coding for an XRE-family Helix-turn-helix protein) was identified in pGN28-KPC, again causing the hypothetical gene *orf393* to become a pseudogene.

**FIGURE 2:**
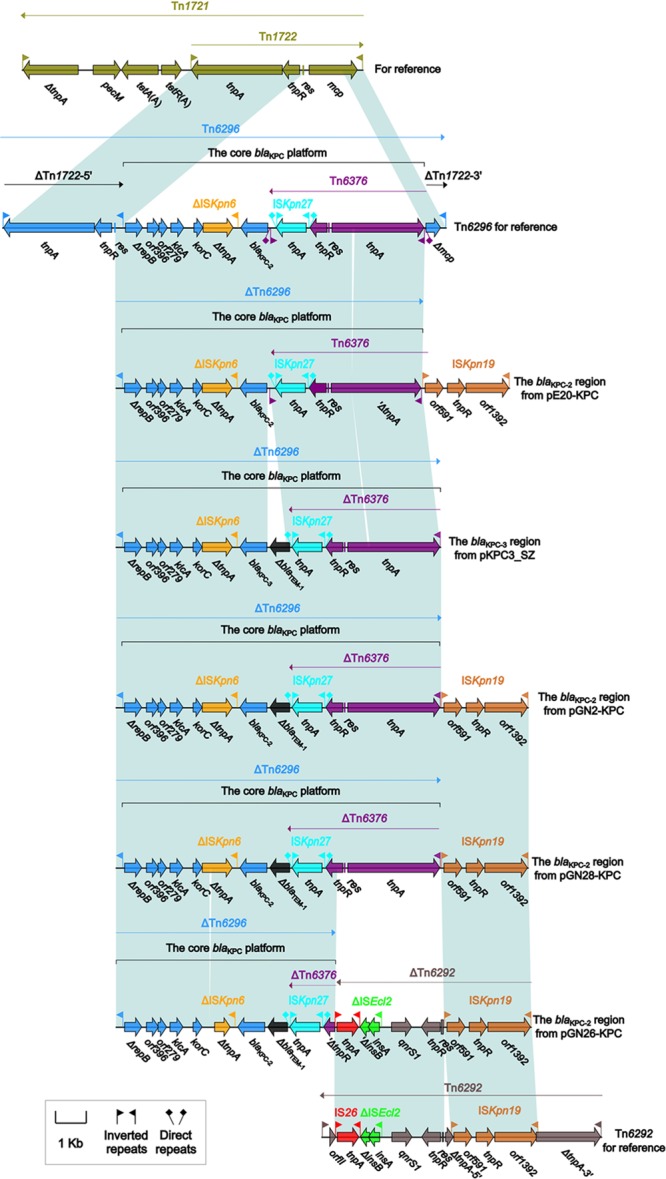
The *bla*_KPC_ regions from IncX6 plasmids, and comparison with related regions. Genes are denoted by arrows. Genes, mobile elements, and other features are colored based on function classification. Shading denotes regions of homology (>95% nucleotide identity).

The accessory modules of pGN2-KPC, pGN26-KPC, pGN28-KPC, pE20-KPC, and pKPC3_SZ were named the *bla*_KPC_ regions (**Figure 1**), and were highly similar to one another (**Figure 2**). The *bla*_KPC_ regions from pGN2-KPC, pGN28-KPC, and pE20-KPC comprised a ΔTn*6296* derivative and an IS*Kpn19* element, while that from pGN26-KPC consisted of a ΔTn*6296* derivative and an IS*Kpn19*-containing ΔTn*6292* derivative. The *bla*_KPC_ region from pKPC3_SZ contained only a ΔTn*6296* derivative (**Figure 2**).

Tn*6296* was originally identified in plasmid pKP048 from *K. pneumoniae* (Jiang et al., 2010). It was generated by the insertion of the core *bla*_KPC-2_ genetic platform (Tn*6376*–*bla*_KPC-2_–ΔIS*Kpn6*–*korC*–*orf6*–*klcA*–Δ*repB*) into the *mcp* (methyl-accepting chemotaxis protein) gene of the cryptic transposon Tn*1722*, truncating *mcp* and splitting Tn*1722* into ΔTn*1722-5′* [IRL (inverted repeat left)–*tnpAR*–*res*] and ΔTn*1722-3′* [*Δmcp*–IRR (inverted repeat right)]. The Tn*3*-family unit transposon Tn*6292*, as observed in pIMP-HZ1 from *K. pneumoniae*, carried a core quinolone resistance genetic platform, *qnrS1*-ΔIS*Ecl2*, and contained an IS*Kpn19* insertion within *tnpA* (Feng et al., 2016; Liang et al., 2017).

The ΔTn*6296* derivatives from these five plasmids were slightly different from each other, with deletions and insertions relative to the prototype Tn*6296* (**Figure 2** and **Supplementary Table S2**). First, ΔTn*1722-5′* was lost from all five ΔTn*6296* elements. Second, a 70-bp deletion within *tnpA* of Tn*6376* and a 70-bp deletion within Δ*tnpA* of ΔIS*Kpn6* were found in pE20-KPC and pGN26-KPC, respectively, leading to frameshift mutations of these two coding regions. Third, the insertion of a 624-bp Δ*bla*_TEM-1_-containing region between IS*Kpn27* and *bla*_KPC-2/3_ was identified in pGN26-KPC, pGN2-KPC, pGN28-KPC, and pKPC3_SZ. Two promoters, consisting of the intrinsic P1 promoter and an upstream Tn*6376*-provided P2 promoter, were found to govern the *bla*_KPC-2_ expression of Tn*6296* (Wang et al., 2015). The insertion of the Δ*bla*_TEM-1_-containing region resulted in the loss of IRL_Tn_*_6376_* and P1 (**Supplementary Figure S2**), leaving P2 as the only promoter for *bla*_KPC_ expression. Finally, the 3′-terminal regions of these five ΔTn*6296* derivatives were truncated in different formats: (i) ΔTn*1722-3′* was absent in each of pE20-KPC, pGN2-KPC, and pGN28-KPC due to connection of IS*Kpn19*; (ii) in pGN26-KPC, the *qnrS1*-containing ΔTn*6292* element (6.4 kb in length) was connected with a 1.6-kb ΔTn*6376* remnant with deletion of IRL–*tnpA*–*res* and truncation of *tnpR*, and the introduction of ΔTn*6292* into pGN26-KPC probably resulted from homologous recombination between Tn*6292* and a pre-existing IS*Kpn19* element (as observed in pE20-KPC, pGN2-KPC, and pGN28-KPC), with IS*Kpn19* acting as the common region necessary for recombination; and (iii) ΔTn*1722-3’* was also lost in pKPC3_SZ, although none of the IS*Kpn19*-related elements or any other regions were found to be adjacent to the 3′-end of ΔTn*6296*.

In summary, complex transposition and homologous recombination events, particularly those involving the three prototype mobile elements Tn*6296*, Tn*6292*, and IS*Kpn19*, occurred to promote the assembly and mobilization of the *bla*_KPC_ regions in these plasmids.

## Discussion

IncX6 plasmid backbones have very limited sequence identity (<18% BLAST coverage and <84% nucleotide sequence identity) to those of other subgroups. Indeed, dramatic genetic diversities are presented among different IncX subgroups. Nevertheless, IncX6 plasmids contain the core IncX backbone makers responsible for plasmid replication initiation (*repA* and *bis*), maintenance (*parA, hns*–*hha*–*topB, relEB*, and *dnaJ*), and conjugal transfer (*rlx, dtr, tivB, cpl, eex*, and *actX*).

Previously sequenced IncX plasmids mostly belong to the IncX1–IncX4 subgroups, with very few representatives of IncX5–IncX7 plasmids. Currently, only five IncX6 plasmids have been fully sequenced, including pKPC3_SZ (Du et al., 2016) and the pGN2-KPC, pGN26-KPC, pGN28-KPC, and pE20-KPC plasmids sequenced in the current study. The five plasmids all originate from clinical isolates belonging to different *Enterobacteriaceae* species, namely *E. cloacae, P. mirabilis, S. marcescens, M. morganii*, and *K. aerogenes*, respectively, all of which come from China. Each of these five IncX6 plasmids contains a single accessory module containing two or three resistance genes, with all five carrying *bla*_KPC-2/3_, pKPC3_SZ, pGN2-KPC, and pGN28-KPC harboring *Δbla*_TEM-1_, and pGN26-KPC containing both *Δbla*_TEM-1_ and *qnrS1*. None of these IncX6 plasmids encodes multi-drug resistance. IncX6 plasmids appear to be an important vehicle for *bla*_KPC_ genes in China, and the core genetic environments of *bla*_KPC_ genes are close derivatives of Tn*6296*.

pGN2-KPC, pGN26-KPC, and pGN28-KPC were the only plasmids detected in their corresponding host strains. pKPC3_SZ coexists with pNDM1_SZ1, a multidrug resistance IncC plasmid that carries genes contributing to resistance to carbapenems (*bla*_NDM-1_), macrolides [*mph*(E)], aminoglycosides (*strAB* and *addA2*), and sulphonamides (*sul1* and *sul2*) (Du et al., 2016). pE20-KPC coexists with four additional plasmids, including pE20-FIIA (GenBank accession number MG288681; IncFII), pE20-HI3 (GenBank accession number MG288682; IncHI3), pE20-NR (GenBank accession number MG288683), and pE20-qnrS (GenBank accession number MG288684) belonging to two unknown incompatibility groups [GenBank accessions not yet released]. Other than pE20-NR, the plasmids that co-exist with the IncX6 plasmid described in the current study all confer resistance to one or more antimicrobial agents, and mediate resistance to at least seven classes of antibiotics (β-lactams including carbapenems, quinolones, macrolides, aminoglycosides, amphenicols, sulphonamides, and trimethoprims). This severely limits the choice of antibiotics for treatment of infections caused by these bacterial strains.

Genomic and epidemiological analyses herein show that *bla*_KPC_-carrying IncX6 plasmids are present in 44.4% of the analyzed *bla*_KPC_-positive isolates from a single hospital and have disseminated among at least six different *Enterobacteriaceae* species from six distinct departments of this hospital, indicating wide spread of these plasmids in this hospital. Further studies are needed to determine the prevalence of IncX6 plasmids among various geographic areas to understand the contribution of IncX6 plasmids to *bla*_KPC_ epidemiology among *Enterobacteriaceae* isolates.

## Author Contributions

DZ and BoL: conception and design of the study. BiL, JF, DZ, BoL, ZZ, ZY, QJ, PW, XC, BG, JH, PM, WW, WC, YT and JW: acquisition of data. BiL, JF, DZ, and BoL: analysis and interpretation of data. BiL, JF, DZ, and BoL: drafting the article. BiL, JF, DZ, BoL, ZZ, ZY, QJ, PW, XC, BG, JH, PM, WW, WC, YT, and JW: critical revision. All authors read and approved the final manuscript.

## Conflict of Interest Statement

The authors declare that the research was conducted in the absence of any commercial or financial relationships that could be construed as a potential conflict of interest.

## Footnotes

1 https://www.ncbi.nlm.nih.gov/pathogens/beta-lactamase-data-resources/

2 https://github.com/ruanjue/smartdenovo

3 https://inkscape.org/en/

## Supplementary Material

FIGURE S1 Schematic maps of IncX6 plasmids. Genes are denoted by arrows, and the backbone and accessory module regions are highlighted in black and gray, respectively. The innermost circle presents GC-skew [(G-C)/(G+C)], with a window size of 500 bp and a step size of 20 bp. The next-to-innermost circle presents GC content.

FIGURE S2 Alignment of promoter-proximal regions of *bla*_KPC-2_. The 898–354 bp upstream sequences together with the start codon of the *bla*_KPC-2_ genes from IncX6 plasmids and Tn*6296* are aligned by MUSCLE. Shown are core promoter regions, -35 and -10 elements, transcription starts, Shine-Dalgarno (SD) sequences for ribosome recognition and translation starts.

TABLE S1 KPC-producing *Enterobacteriaceae* strains.

TABLE S2 Major features of ΔTn*6296* derivatives compared to Tn*6296.*
