# Supplementary figures and images for: Dissemination of KPC-2-Encoding IncX6 Plasmids Among Multiple Enterobacteriaceae Species in a Single Chinese Hospital
Source: Front Microbiol. 2018 Mar 19;9:478. doi: 10.3389/fmicb.2018.00478 (PMC5868456; doi:10.3389/fmicb.2018.00478)

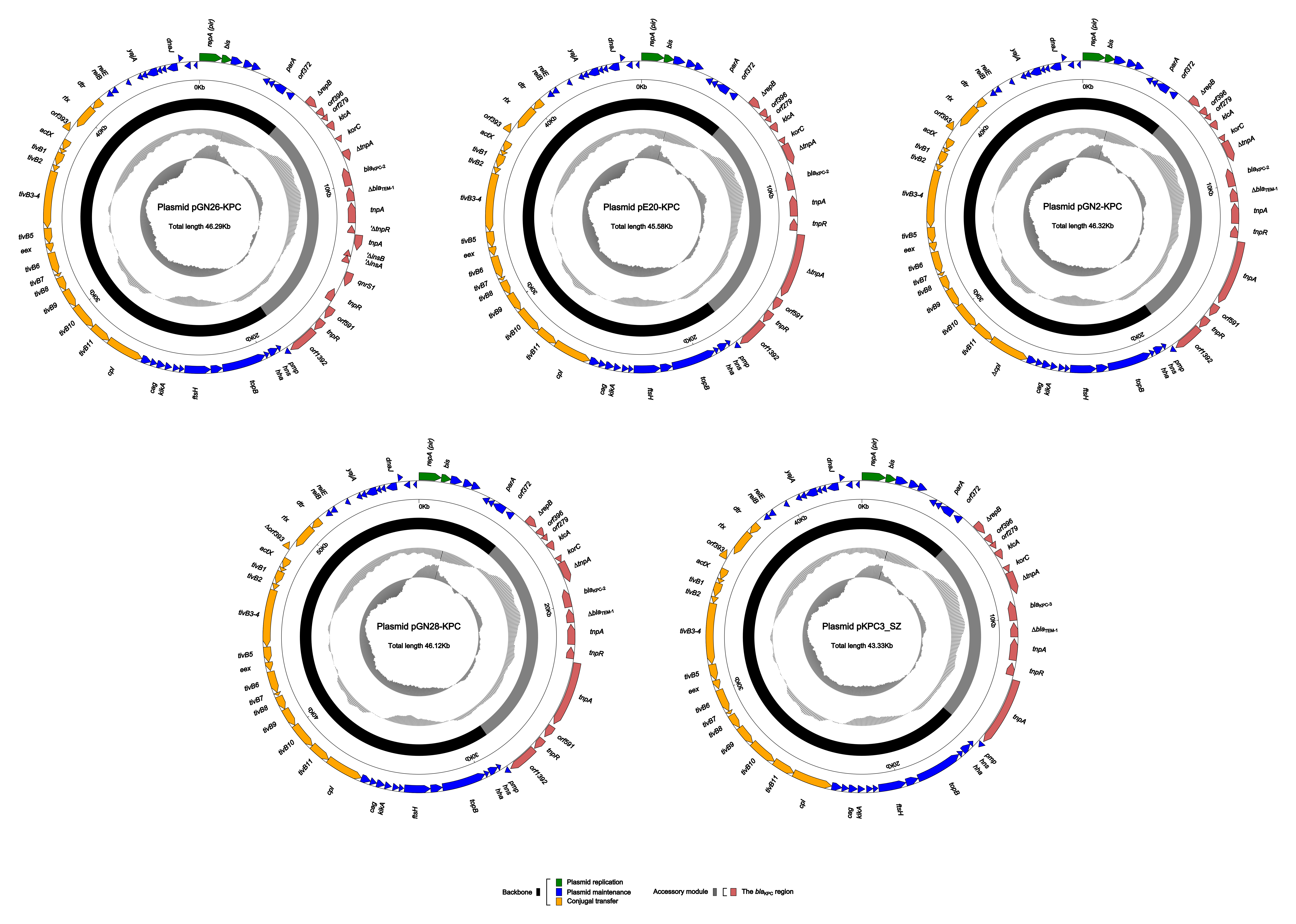

Supplement: FIGURE S1 — Schematic maps of IncX6 plasmids. Genes are denoted by arrows, and the backbone and accessory module regions are highlighted in black and gray, respectively. The innermost circle presents GC-skew [(G-C)/(G+C)], with a window size of 500 bp and a step size of 20 bp. The next-to-innermost circle presents GC content. [file Image_1.TIF]

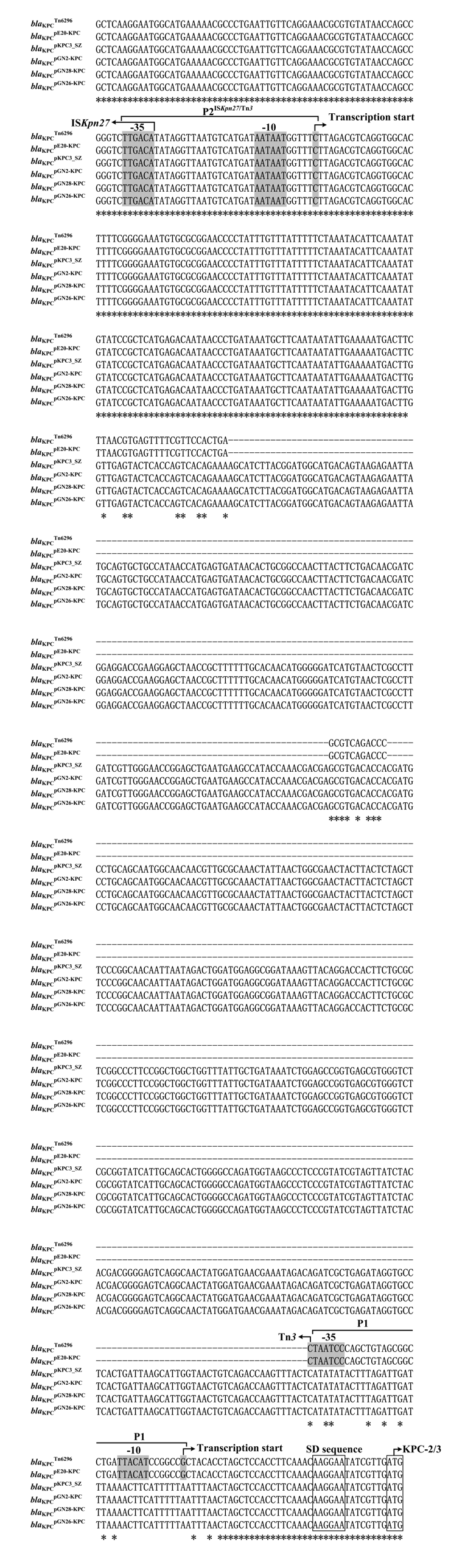

Supplement: FIGURE S2 — Alignment of promoter-proximal regions of blaKPC-2. The 898–354 bp upstream sequences together with the start codon of the blaKPC-2 genes from IncX6 plasmids and Tn6296 are aligned by MUSCLE. Shown are core promoter regions, -35 and -10 elements, transcription starts, Shine-Dalgarno (SD) sequences for ribosome recognition and translation starts. [file Image_2.TIF]
