# Supplementary material for: Dissemination of KPC-2-Encoding IncX6 Plasmids Among Multiple Enterobacteriaceae Species in a Single Chinese Hospital
Source: Front Microbiol. 2018 Mar 19;9:478. doi: 10.3389/fmicb.2018.00478 (PMC5868456; doi:10.3389/fmicb.2018.00478)
Supplement: TABLE S2 — Major features of ΔTn6296 derivatives compared to Tn6296. [file Table_2.DOCX]

**Table S2 Major features of ΔTn*6296* derivatives compared to Tn*6296***

| Plasmid | Deletions | Insertion |
| --- | --- | --- |
| pE20-KPC | ΔTn*1722*-5', a 70-bp deletion within *tnpA* of Tn*6376*, and ΔTn*1722*-3' | None |
| pKPC3_SZ | ΔTn*1722*-5' and ΔTn*1722*-3' | A 624-bp insertion (Δ*bla*_TEM-1_-containing region) between IS*Kpn27* and *bla*_KPC-2/3_ |
| pGN2-KPC | ΔTn*1722*-5' and ΔTn*1722*-3' | A 624-bp insertion (Δ*bla*_TEM-1_-containing region) between IS*Kpn27* and *bla*_KPC-2/3_ |
| pGN28-KPC | ΔTn*1722*-5' and ΔTn*1722*-3' | A 624-bp insertion (Δ*bla*_TEM-1_-containing region) between IS*Kpn27* and *bla*_KPC-2/3_ |
| pGN26-KPC | ΔTn*1722*-5', a 70-bp deletion within Δ*tnpA* of ΔIS*Kpn6*, IRL–*tnpA*–*res*–*tnpR*-5' of ΔTn*6376*, and ΔTn*1722*-3' | A 624-bp insertion (Δ*bla*_TEM-1_-containing region) between IS*Kpn27* and *bla*_KPC-2/3_ |
